# A spatiotemporal analysis of opioid poisoning mortality in Ohio from 2010 to 2016

**DOI:** 10.1038/s41598-021-83544-y

**Published:** 2021-02-25

**Authors:** Chihyun Park, Jean R. Clemenceau, Anna Seballos, Sara Crawford, Rocio Lopez, Tyler Coy, Gowtham Atluri, Tae Hyun Hwang

**Affiliations:** 1 Department of Quantitative Health Sciences (QHS), Lerner Research Institute, Cleveland Clinic, 9500 Euclid Ave, Cleveland, OH 44195 USA; 2 Department of Computer Science and Engineering, Kangwon National University, Chuncheon, Republic of Korea; 3 Department of Electrical Engineering and Computer Science (EECS), University of Cincinnati, P.O. Box 210030, Cincinnati, OH 45221 USA

**Keywords:** Epidemiology, Population screening

## Abstract

Opioid-related deaths have severely increased since 2000 in the United States. This crisis has been declared a public health emergency, and among the most affected states is Ohio. We used statewide vital statistic data from the Ohio Department of Health (ODH) and demographics data from the U.S. Census Bureau to analyze opioid-related mortality from 2010 to 2016. We focused on the characterization of the demographics from the population of opioid-related fatalities, spatiotemporal pattern analysis using Moran’s statistics at the census-tract level, and comorbidity analysis using frequent itemset mining and association rule mining. We found higher rates of opioid-related deaths in white males aged 25–54 compared to the rest of Ohioans. Deaths tended to increasingly cluster around Cleveland, Columbus and Cincinnati and away from rural regions as time progressed. We also found relatively high co-occurrence of cardiovascular disease, anxiety or drug abuse history, with opioid-related mortality. Our results demonstrate that state-wide spatiotemporal and comorbidity analysis of the opioid epidemic could provide novel insights into how the demographic characteristics, spatiotemporal factors, and/or health conditions may be associated with opioid-related deaths in the state of Ohio.

## Introduction

Opioids are a class of drugs derived from the opium poppy plant and have the therapeutic but potentially addictive effect of blocking the reception of pain signals in the brain. Opioids include prescription pain relievers such as oxycodone, hydrocodone, codeine, and morphine, which are used to alleviate chronic pain and manage postoperative pain. In addition to their medical use, some opioids are also used illegally, particularly heroin and synthetic opioids such as fentanyl. While the number of opioid prescriptions written skyrocketed in the last two decades, illegal opioid use, opioid abuse and accidental opioid overdoses have increased at an even greater rate^1^. Opioid-related overdoses are commonly referred to and categorized as “opioid poisoning” according to International Classification of Disease (ICD) codes (ICD-10 codes: T40.0-T40.4, T40.6)^2^. The mortality rate due to opioid poisoning has consistently increased each year and now it is recognized as a national crisis^3^. Since 2000, deaths from drug overdoses have increased by 137%, while deaths from opioid overdoses have increased by 200%^4^. According to the data released by the U.S Department of Health and Human Services, 130 people died each day from opioid-related drug overdoses in 2017 and 11.5 million people misused prescription opioids^5^.

The opioid epidemic is a nationwide issue, and scientific research is key to identifying causal factors, discovering susceptible communities and developing policies to address this problem. Increased availability of healthcare data presents a tremendous opportunity for data analysis to identify factors associated with opioid misuse and comorbidities associated with deaths due to opioid poisoning^6^. There have been several attempts to apply computational or statistical analysis to inform solutions for the problems associated with the opioid epidemic^7–11^. The purposes of these studies varied, ranging from understanding and detecting geographical clusters or hot spots where opioid misuse has occurred within urban neighborhoods^12^, to developing a surveillance model to identify patients who are misusing opioids or are over-prescribed^10^. Spatiotemporal analysis has been applied to identify relationships between environmental or economic factors and opioid misuse or overdose^12–15^. Through these attempts, several covariates associated with opioid misuse or overdose have been found. Housing vacancy, dilapidated housing and misdemeanor arrests have been shown to be associated with illicit drug activity^12,13^. Additionally, higher income and greater access to healthcare have been found to be associated with prescription opioid poisoning^14^. Existing research on comorbidity with opioid abuse focuses mostly on psychiatric disorders and co-occurring substance abuse^7,9,15–18^. Logistic regression models have been developed to identify characteristics of patients susceptible to prescription opioid abuse^7–9^. Comorbid psychiatric disorders have been considered for their impact on the efficacy of various treatments for opioid use disorder^15^, and literature reviews have found high rates of co-occurrence between opioid use disorder and anxiety, psychiatric comorbidity, and other drug use^16–18^.

These studies focus on either city-level spatiotemporal patterns, and/or solely on abuse of a single class of opioids. Our research performs demographic disparity analysis, spatial pattern mining, and comorbidity analysis on a large-sized dataset collected at a state-wide level for all opioid-related deaths. Our research utilizes a large-scale vital statistic dataset from the Ohio Department of Health (ODH) and the U.S. Census Bureau to perform retrospective analysis at a statewide and census tract level. The purpose of this study is to identify demographic and spatial factors, as well as co-occurring health conditions that are associated with opioid-related overdose. To achieve this goal, we applied several data mining approaches such as spatial clustering and frequent pattern mining. We expect that our methodology and results will help inform policy makers, law enforcement, emergency health services, and caregivers to prevent opioid abuse throughout Ohio, and help inform the development of predictive models in future works.

## Methods

Mortality data were collected from the ODH Bureau of Vital Statistics, through the Ohio Public Health Information Warehouse. The Department specifically disclaims responsibility for any analyses, interpretations, or conclusions from these data. The data contain demographic and clinical information about victims of fatalities caused by opioid poisoning and misuse from 2010 to 2016. This study did not require Institutional Review Board approval and informed consent was not necessary since we analyzed records of deceased individuals in Ohio, which are not considered human subjects under the U.S. Department of Health & Human Services Common Rule Issues No. 45 CFR § 46.102^19^ and death records are publically available in Ohio. Our methods are consistent with the ethical guidelines from the Declaration of Helsinki. We de-identified and filtered ODH mortality data by selecting only opioid-related records. We considered an opioid-related death (ORD) to be a death record with at least one of the following terms or term strings: Methadone, opiates, prescription opiates, Fentanyl, Fentanyl and Analogues, Carfentanil, “designer opioids”, “commonly prescribed opioids”, or “other opioids”. Then, from these ORDs we chose records for analysis whose free text contained the strings: “Unintended” or “Undetermined” for the “ExternalInjuryIntent” column, and “Drug Poisoning” in the “ExternalInjuryMechanism” column. As a result, 13,094 records satisfied these conditions. Of these records, 13,057 were not missing any values in the demographic and location fields. We used these filtered records for our analyses. This dataset is hereafter referred to as “ODH-Opioid data.” Supplemental Fig. S1 shows the entire process of data refinement.

To gather characteristics of the entire population of Ohio, as well as from each Ohio census tract, we obtained publicly available demographic data from the 2010 U.S. Census Bureau and from the American Community Survey (ACS) for the years 2011 to 2016.

### Demographic-based analysis to survey disparities among population groups

Decedent demographic information, such as age, race and sex, was taken from ODH-Opioid data. The same population-level demographic data in Ohio were then obtained from the ACS. We compared and analyzed the frequency of demographic characteristics in ORD records relative to their frequency in the overall population. We calculated relative proportions for each population group based on these two datasets for seven years. Then, we performed a paired t-test to investigate the statistical difference between the proportions of each demographic variable in the ORD and general populations.

### Spatiotemporal pattern analysis at the census tract level to discover potential global and local spatial clusters

Each record in ODH-Opioid data contains geographical information about a given decedent, such as residence or place of death. It is necessary to investigate whether deaths tend to be focused or dispersed in a specific region to identify spatial patterns of opioid abuse. To achieve this spatial understanding, we applied spatial autocorrelation statistics. Moran statistics are one of the commonly used measures of spatial autocorrelation^20^.

A Moran’s I statistics value close to + 1 indicates that objects with similar properties are spatially clustered (i.e. have strong positive spatial autocorrelation). Otherwise, if Moran’s I statistics value is close to -1, the spatial objects are perfectly scattered or dispersed (like a checkerboard pattern). Near-perfect scattering indicates a strong negative spatial autocorrelation. If the Moran’s I statistic value is close to 0, the objects are randomly ordered in space. This randomness means there is no spatial autocorrelation.

There are two types of Moran’s *I* statistics: global and local. Global Moran’s *I* statistics provide a single measure of spatial autocorrelation for an attribute in a region as a whole. Local Moran’s *I* statistics provide a measure of the tendency of a given region to have an attribute value that is correlated with values of nearby areas. We utilized the Local Indicators of Spatial Autocorrelation (LISA) statistic, a method based on Local Moran’s I statistics commonly used for the identification of local patterns of spatial association.

In our analysis, we used census tract as the unit of region. Census tracts are small, relatively permanent statistical subdivisions within a county^21^. We aggregated the number of deaths by census tract and then normalized the number of deaths by dividing by the total population of the corresponding tract. This normalization allowed spatial autocorrelation to be performed on the ratios of tract death counts to tract population. We used these census tract death rates for spatiotemporal pattern analysis.

We performed spatiotemporal pattern analysis by using a free, open source software tool, GeoDa version 1.12.1.129 (https://geodacenter.github.io/download.html. ^22^ We applied Global Moran’s *I* statistics to Ohio at the state-level and LISA statistics to each region by census tract. GeoDa output also provides two-tailed p-values to show statistical significance, and we used the default alpha value of 0.05.

### Analysis of comorbidity associations with cause of death

This analysis aimed to identify which health conditions frequently co-occurred with opioid poisoning and whether the records of ORD victims exhibited significant associations with such conditions. We applied frequent itemset mining and association rule mining to ODH-Opioid data to carry out these analyses^23^. Generally, the goal of frequent itemset mining is to identify patterns of frequently co-occurring items within a dataset. For example, in a given set of items in a database of transactions, all subsets of items that occur together in many transactions are determined, and their frequency of occurrence establishes their support count. If the frequent itemsets fulfill a minimum support count, in our case the minimum support count is 200, then association rules can be generated. Association rules correlate the presence of one set of items with that of another set of items in the transaction database. These rules must satisfy minimum support and confidence parameters.

Although ODH-Opioid data are filtered as “death by opioid misuse,” the specific causes of death are more diverse. In the ODH mortality dataset, the “Record-Axis Codes” (RAC) field listed all ICD codes (ICD-9 and ICD-10) associated with causes of death for an individual. On the other hand, associated health conditions were listed in literal, comma-separated form in the “OtherConditions” field. In order to analyze the observed health conditions, we had to simplify the associated condition’s literal text as reported for the deceased person to a standard term. We vectorized ICD code descriptions using the bag-of-words algorithm^24^ and subsequently used agglomerative clustering (k = 70). We selected the most frequent term from each cluster, and then redundant clusters were manually removed by a physician. Through this post-processing, we obtained 31 representative terms describing a given decedent’s health condition, such as “hypertension” or “drug abuse”. Supplementary Fig. S2 shows the overall workflow for the text clustering method. Then, we integrated cause of death with standardized health condition terms and applied frequent itemset mining to reveal which events (i.e. itemset) frequently occur together.

From the results of our frequent itemset mining, we performed association rule mining with a minimum confidence of 0.5 in order to quantify the association between two itemsets. We sorted all rules by Lift value, which measures how much the association rule occurs compared to what would be expected if the occurrences of the items in question were statistically independent. Once sorted, only the rules with at least one health condition in itemset A and at least one cause of death in itemset B were selected from the results (Lift > 1: positive relationship, which means if A is present, then B tends to be present as well).

## Results

### Demographic-based analysis to survey disparities among population groups

**Figure 1:**
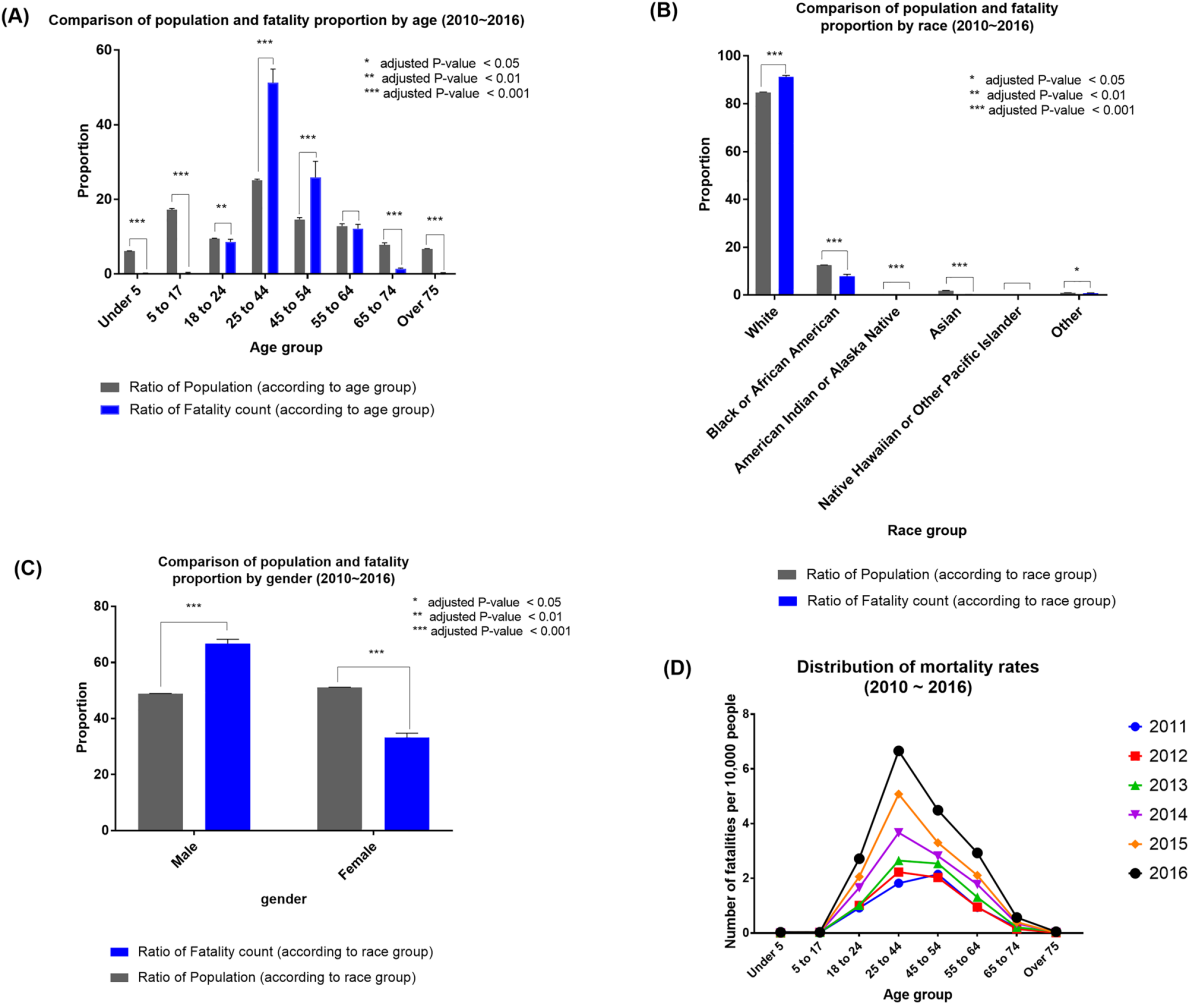
Comparison of population and mortality proportion by several demographic factors.

Using ODH-Opioid data, we compared the demographics between the overall Ohio population and those who died due to opioid poisoning. Figure 1A shows the difference between the age distributions of the general population and the opioid fatality victims for all seven years.. For example, while the group aged 24–44 years made up an average of 25.1% of the overall population across the 7 years, the same age group consisted of an average of 51.3% of the opioid deaths. We performed pairwise t-tests between the two groups for different age groups and found that all described age groups have a significant difference in death proportions (*p* < 0.001) except the 55–64-year age group (*p* = 0.19). In particular, groups aged 25–44 and 45–54 years showed a higher risk of opioid poisoning fatality than any other age group compared to the general population with a mean difference of − 26.14 and − 11.27, respectively. We performed the same analysis for race and sex, shown in Fig. 1B,C, respectively. There was a statistically significant difference identified between the mortality and population distribution for all races (*p* < 0.05) except for “Native Hawaiian or Other Pacific Islander” (*p* = 0.33), with the white population exhibiting the highest mean death count difference (− 6.46). Similarly, there was a statistically significant difference in the distributions for both males and females (*p* < 0.001). Males had a higher mean fatality proportion (− 17.89), while females had a lower mean fatality proportion (17.89) compared to their respective population proportions. In summary, between 2010 and 2016 in Ohio, people who were white, male or aged 25–54 years were most likely to die by opioid poisoning. Supplementary Table S1 shows detailed results of these statistical tests.

Finally, as shown in Fig. 1D, we calculated the mortality rate per 10,000 people for each age group in each year. As we concluded earlier, mortality rates for age groups 25–44 and 45–54 were the highest and the ratio increased with time.

### Discovering global and local spatial clusters using spatiotemporal pattern analysis at census tract level

**Figure 2:**
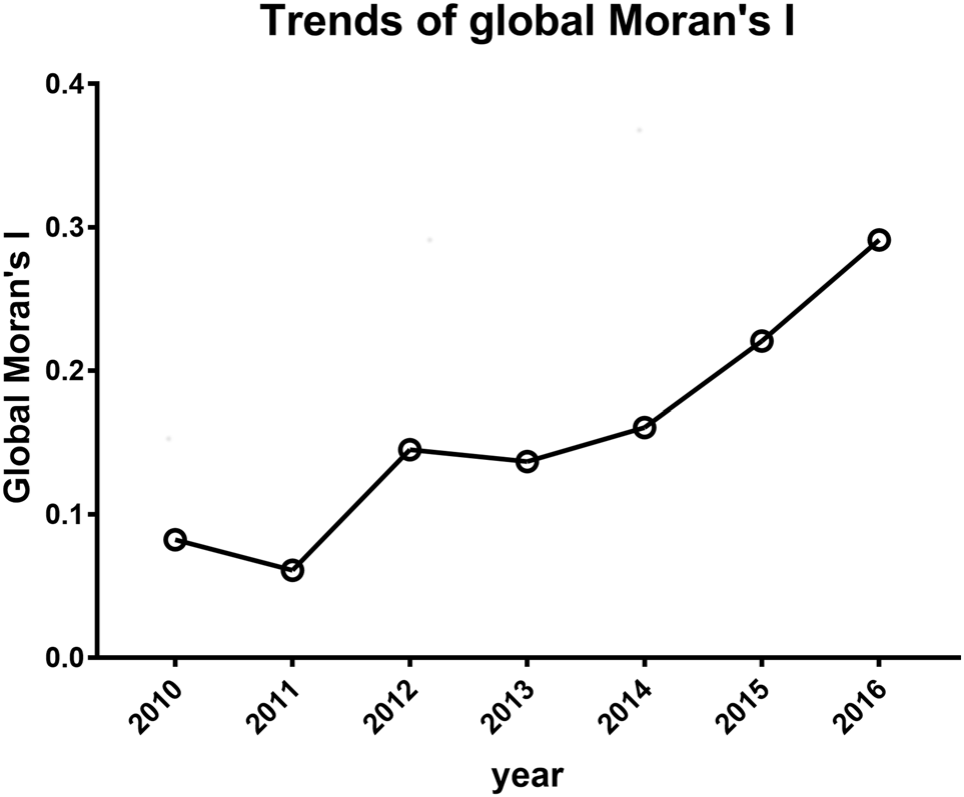
Changing pattern of Global Moran’s I statistics for seven years in Ohio; 2010–2016.

The Global Moran's I statistic provides a single measure of spatial autocorrelation for an attribute in a region as a whole. As seen in Fig. 2, this statistic continuously increased from 2010 to 2016. This increase signifies higher specificity and lower randomization of clustering, meaning that opioid-associated mortalities became more spatially concentrated during these years.

**Figure 3:**
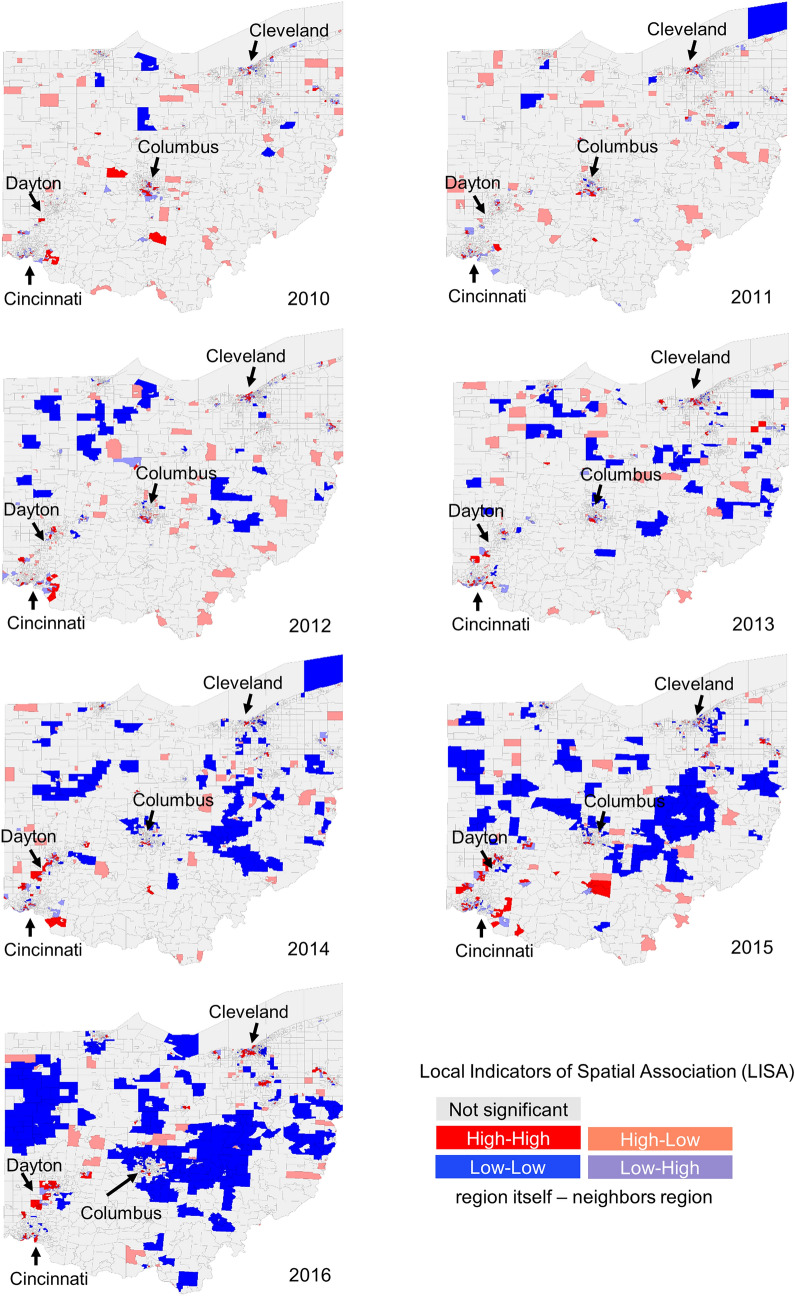
Spatial clusters with normalized mortality (number of deaths divided by number of population) for opioid poisoning by census tract, Ohio, 2010–2016. Figure produced using GeoDa software.

Figure 3 demonstrates the results of our spatiotemporal pattern analysis for each census tract. There are four types of patterns of LISA statistics: High-High, High-Low, Low–High, and Low-Low. The first field of a given LISA pattern indicates the degree of LISA statistics in the corresponding region and the second field indicates the degree of LISA statistics of the neighboring regions. For example, a High-Low designation for a certain region means the LISA statistic of the region is significantly high but the values of its neighboring regions are significantly low. Generally, we can consider High-High and Low-Low status as spatial clusters because these regions have similar LISA statistics values to their neighbors. In other words, a spatial cluster is a collection of adjacent geographical regions with a similar spatial autocorrelation characteristic. Figure 3 demonstrates that the High-High regions become more concentrated, while Low-Low regions increasingly disseminated from 2010 to 2016. We can observe that the census tracts inside or near Cleveland, Columbus, and Cincinnati, which are the most populated cities in Ohio, had increasingly the highest concentration of deaths by opioid poisoning as years advanced, while lower population and rural census tracts tended to have decreasing ORDs. Overall, these results show a shift in ORD occurrence from dispersed and rural to concentrated and urban patterns.

Supplementary Table S2 shows the top 10 census tracts represented by county name based on the LISA statistics value.

### Analysis of comorbidity associations with cause of death

We used health condition and cause of death terms classified by ICD-10 codes as input for frequent itemset and pattern mining to reveal the underlying relationship between health condition and cause of death with regard to opioid poisoning mortality. Of the total 13,094 records, only 2,887 records contained data for both health condition and cause of death, so the remaining records, which had a value of “NA,” were excluded for the co-occurrence analysis. Supplementary Table S3 shows the representative terms for health conditions of the deceased.

Frequent itemset mining was performed as described in the methods section. Table 1 shows the top 10 results based on frequency for this analysis. We observed that cardiovascular disease was the most prevalent concomitant health condition in decedents by opioid poisoning. Cardiovascular disease frequently occurred with death due to hypertensive heart disease without heart failure. Drug abuse frequently occurred with death due to mental and behavioral disorders because of multiple drug use. Additionally, mental health conditions, such as anxiety, were frequently associated with opioid poisoning mortality.

**Table 1:** List of top 10 most frequent results which have more than two items including one or more health conditions in frequent itemset.

Frequent Itemset	Frequency	Count	Description for cause of death	Description for health condition
X42, clu_4	0.168	483	Accidental poisoning by and exposure to narcotics and psychodysleptics [hallucinogens], not elsewhere classified	Cardiovascular disease
I119, clu_4	0.149	429	Hypertensive heart disease without (congestive) heart failure	Cardiovascular disease
T509, clu_4	0.139	400	Poisoning: Other and unspecified drugs, medicaments and biological substances	Cardiovascular disease
T402, clu_4	0.113	325	Poisoning: Other opioids	Cardiovascular disease
T509, clu_23	0.112	324	Poisoning: Other and unspecified drugs, medicaments and biological substances	Anxiety
I250, clu_4	0.110	319	Atherosclerotic cardiovascular disease, so described	Cardiovascular disease
F191, clu_1	0.109	314	Mental and behavioral disorders due to multiple drug use and use of other psychoactive substances: harmful use	Drug abuse
T401, clu_4	0.103	297	Poisoning: Heroin	Cardiovascular disease
X42, clu_1	0.102	295	Accidental poisoning by and exposure to narcotics and psychodysleptics [hallucinogens], not elsewhere classified	Drug abuse
I119, X42, clu_4	0.098	283	Hypertensive heart disease without (congestive) heart failure Accidental poisoning by and exposure to narcotics and psychodysleptics [hallucinogens], not elsewhere classified	Cardiovascular disease

Cardiovascular disease was the most prevalent concomitant health condition in decedents.

Table 2 shows the results of association rule mining. We identified that drug abuse, anxiety and cardiovascular disease were significantly and positively correlated with opioid poisoning mortality.

**Table 2:** List of the selected association rules which contain health condition in itemset A and cause of death in itemset B ordered by Lift value (≥ 1).

Rule: *A* → *B*		Confidence (> 0.5)	Lift (correlation for dependency or independency between A and B)	Detailed description for rules
Itemset *A*	Itemset *B*			
clu_6	I517	0.737	7.161	Cardiomegaly → Cardiomegaly
clu_4	I119	0.542	3.396	Cardiovascular Disease → Hypertensive heart disease without (congestive) heart failure
clu_1	F191	0.631	2.754	Drug abuse → Mental and behavioral disorders due to multiple drug use and use of other psychoactive substances: harmful use
clu_23	T402	0.504	1.364	Anxiety → Poisoning: Other opioids
clu_1	T401	0.502	1.322	Drug abuse → Poisoning: Heroin
clu_23	T509	0.582	1.117	Anxiety → Poisoning: Other and unspecified drugs, medicaments and biological substances
clu_1	T509	0.558	1.072	Drug abuse → Poisoning: Other and unspecified drugs, medicaments and biological substances
clu_4	X42	0.612	1.053	Cardiovascular disease → Accidental poisoning by and exposure to narcotics and psychodysleptics [hallucinogens], not elsewhere classified
clu_1	X42	0.592	1.020	Drug abuse → Accidental poisoning by and exposure to narcotics and psychodysleptics [hallucinogens], not elsewhere classified

## Discussion

The opioid epidemic is one of the most pressing issues affecting Ohio in recent years. This severity was exemplified in August 2019, when six people died of suspected fentanyl overdose within 24 h, bringing the opioid-overdose death count to 10 for a three-day period^23^. All 10 deaths occurred within Cuyahoga County, Ohio, which encompasses Cleveland. This event emphasizes the need to understand the spread and risk factors associated with opioid-related deaths. With access to opioid abuse and overdose data, it is imperative to apply computational analyses to better understand the opioid epidemic.

To the our knowledge, there are currently no large-scale, data-driven approaches covering demographic disparity profiling, spatial pattern mining, and association rule mining with statewide mortality records for all opioid-related deaths throughout the state of Ohio. Our research provides a sweeping analysis in these areas, showing that various data mining algorithms such as text mining, spatial clustering and frequent pattern mining can be used to effectively investigate the current situation of the opioid overdose epidemic. The opioid epidemic is an important subject for research in the public health field, and a data science approach could contribute to a more complete understanding of the issue. Data-driven analyses have potential to expose previously unknown factors of value for the mitigation of this epidemic.

The first approach of our three-pronged analysis explored the incidence of opioid abuse among population groups in order to find death disparities. We found that people aged 25–44 and 45–54 were at a higher risk of having an ORD than any other age group, and that whites and males also had higher rates of death due to opioid overdose than other groups. Our second approach used spatial autocorrelation to map geographical patterns of opioid abuse, and we found that opioid-related deaths have become more spatially concentrated over time. In particular, Cleveland, Columbus and Cincinnati had the highest concentration of deaths. Finally, our third approach focused on identifying health conditions that co-occurred with opioid-related death. We found that drug abuse, anxiety and cardiovascular disease were significantly and positively correlated with death due to opioid overdose.

The increasing spatial concentration of opioid overdose deaths that we observed is an interesting finding. From 1999 to 2004, opioid-related death rates in rural areas exceeded those in urban areas^25^, but recent data from the Centers for Disease Control and Prevention (CDC) show an increase of drug overdose deaths in urban areas compared to that of rural areas in 2017^26^. Our results are consistent with this national pattern, which may suggest that Ohio undergoes similar changes in the spatial distribution of opioid abuse. These analyses could serve as a basis for developing accurate predictive geographical models in the future, which could be essential in targeting prevention strategies for specific regions, as well as allocating resources to areas most in need.

Our comorbidity findings are consistent with existing studies that have identified anxiety, mental illness, and drug use to be associated with opioid abuse^7,8,16–18^. However, to our knowledge, other studies have not found an association between opioid abuse deaths and cardiovascular disease. This finding should be investigated further to determine if there is a significant difference in cardiovascular disease for people who suffered an ORD compared to overall deaths in Ohio. In future studies, we plan to collect more robust data by combining vital records from ODH data and patient Electronic Health Record (EHR) data at the Cleveland Clinic. We recommend that similar studies be performed with data from hospitals in Columbus and Cincinnati to understand other epidemiological and regional patterns. Such analyses could be used to develop a model to stratify patient risk based on previously identified predictors of opioid misuse or death, which could aid caregivers in prescription decision-making.

Furthermore, we suggest specific application of spatial pattern mining to be used to identify areas of opioid abuse real-time, rather than solely in a retrospective fashion. A model able to project which areas are susceptible to opioid-related fatalities could be indispensable for law enforcement agents and emergency health service providers to allocate resources and prevent opioid abuse or death.

As data related to opioid abuse and overdose have recently become available, large-scale, computational approaches to analysis are a crucial tool to combat the opioid epidemic. In this study, we utilized seven years of Ohio vital statistics data to reveal the race, age group, and regional differences of opioid poisoning. Additionally, we identified several prevalent health conditions that are frequently associated with opioid-related deaths in Ohio. Although the data we utilized were retrospective and limited to a specific time window in Ohio, our findings help to understand the patterns and factors associated with opioid-related deaths, which could be useful for authorities and health professionals to monitor populations at risk and for the development of predictive models in the future. Finally, our plan for continuing this work is to include data from 2017 and beyond, as well as more detailed comorbidity information to validate our findings and monitor the contemporary trends of opioid abuse and opioid-related death.

## Limitations

An important limitation of our study was the use of retrospective data from 2010 to 2016. Thus, our study may not capture the more recent influence of newer synthetic opioids such as fentanyl in our analysis. In addition, comorbidity data collected from death records are not available in most cases and counties (e.g., 22% of our data contained information about the health conditions), so our reported results should be interpreted with such consideration in mind and need to be further validated with more complete comorbidity data.

## Conclusion

In this study, statewide vital statistic data from the ODH and demographics data from the U.S. Census Bureau were used to analyze opioid-related mortality from 2010 to 2016. Through various computational analysis, we described the race, age group, and regional disparities of opioid poisoning in the state of Ohio. Additionally, we identified several prevalent pre-existing health conditions that are frequently associated with opioid-related fatalities. Although the data we utilized were limited to seven years in Ohio and our analysis was retrospective in nature, our findings help to understand the patterns and factors associated with opioid-related fatalities.

## Supplementary Information

Supplementary Information 1.

## Data Availability

The datasets analyzed in the current study are publically available from the following sources: 2010 U.S. Census: https://factfinder.census.gov/faces/nav/jsf/pages/download_center.xhtml. 2011–2016 American Community Survey: https://www.census.gov/programs-surveys/acs/data.html. Ohio Department of Health Vital Statistics: http://publicapps.odh.ohio.gov/EDW/DataBrowser/Browse/Mortality.
