# Supplementary material for: A spatiotemporal analysis of opioid poisoning mortality in Ohio from 2010 to 2016
Source: Sci Rep. 2021 Feb 25;11:4692. doi: 10.1038/s41598-021-83544-y (PMC7907120; doi:10.1038/s41598-021-83544-y)
Supplement: Supplementary file 1 — Supplementary Information 1. [file 41598_2021_83544_MOESM1_ESM.pdf]

# Comprehensive Spatiotemporal Analysis of Opioid Poisoning Mortality in Ohio from 2010 to 2016

## Supplementary Material


Chihyun Park[1,2+], Jean R. Clemenceau[1+], Anna Seballos[1], Sara Crawford[1], Rocio Lopez[1], Tyler Coy[1], Gowtham Atluri[3*], Tae Hyun Hwang[1*]

1. Department of Quantitative Health Sciences (QHS), Lerner Research Institute, Cleveland Clinic, Cleveland, Ohio, USA
2. Department of Computer Science and Engineering, Kangwon National University, Republic of Korea
3. Department of Electrical Engineering and Computer Science (EECS), University of Cincinnati

[+]these authors contributed equally to this work

*Corresponding authors:

Gowtham Atluri, Dept. of EECS, University of Cincinnati, P.O. Box 210030, Cincinnati, OH 45221, USA; gowtham.atluri@uc.edu

Tae Hyun Hwang, PhD, Dept. of QHS, Lerner Research Institute, Cleveland Clinic, 9500 Euclid Ave, Cleveland, OH 44195, USA; (216)-442-5565; hwangt@ccf.org



Keywords: opioid, analgesics, socioeconomic, spatiotemporal

| Category | Group | Significant? | Mean1 | Mean2 | Difference | SE of difference | t ratio | Adjusted P Value |
|---|---|---|---|---|---|---|---|---|
| Age | Under 5 | Yes | 6.11 | 0.13 | 5.99 | 0.07 | 90.72 | <0.000000000000001 |
| | 5 to 17 | Yes | 17.24 | 0.29 | 16.96 | 0.14 | 118.10 | <0.000000000000001 |
| | 18 to 24 | Yes | 9.53 | 8.64 | 0.89 | 0.25 | 3.54 | 0.008147083 |
| | 25 to 44 | Yes | 25.13 | 51.27 | -26.14 | 1.40 | 18.72 | 1.20224E-09 |
| | 45 to 54 | Yes | 14.63 | 25.90 | -11.27 | 1.65 | 6.85 | 5.34781E-05 |
| | 55 to 64 | No | 12.84 | 12.17 | 0.67 | 0.48 | 1.39 | 0.190856629 |
| | 65 to 74 | Yes | 7.79 | 1.40 | 6.39 | 0.24 | 27.00 | 2.045E-11 |
| | Over 75 | Yes | 6.74 | 0.19 | 6.56 | 0.07 | 98.61 | <0.000000000000001 |
| Race | White | Yes | 84.71 | 91.17 | -6.46 | 0.28 | 23.31 | 1.15872E-10 |
| | Black or African American | Yes | 12.43 | 7.91 | 4.51 | 0.29 | 15.41 | 1.13898E-08 |
| | American Indian or Alaska Native | Yes | 0.20 | 0.09 | 0.11 | 0.01 | 8.00 | 1.12797E-05 |
| | Asian | Yes | 1.79 | 0.17 | 1.61 | 0.06 | 27.68 | 1.8327E-11 |
| | Native Hawaiian or Other Pacific Islander | No | 0.00 | 0.01 | -0.01 | 0.01 | 1.00 | 0.337049058 |
| | Other | No | 0.86 | 0.69 | 0.17 | 0.07 | 2.58 | 0.047787177 |
| Sex | Male | Yes | 48.87 | 66.76 | -17.89 | 0.59 | 30.41 | 2.007E-12 |
| | Female | Yes | 51.13 | 33.24 | 17.89 | 0.59 | 30.41 | 2.007E-12 |

**Table S 1. Detailed results of the statistical tests for investigating disparities between population groups.**

| Year | 2010 | 2011 | 2012 | 2013 | 2014 | 2015 | 2016 |
|---|---|---|---|---|---|---|---|
| Representing with County for top 10 Tract by LISA | Hamilton | Hamilton | Hamilton | Cuyahoga | Butler | Butler | Summit |
| | Franklin | Hamilton | Cuyahoga | Butler | Butler | Butler | Montgomery |
| | Franklin | Cuyahoga | Montgomery | Butler | Butler | Hamilton | Cuyahoga |
| | Cuyahoga | Hamilton | Franklin | Cuyahoga | Hamilton | Butler | Montgomery |
| | Franklin | Hamilton | Montgomery | Cuyahoga | Butler | Clark | Summit |
| | Ross | Franklin | Cuyahoga | Butler | Butler | Montgomery | Summit |
| | Franklin | Franklin | Cuyahoga | Cuyahoga | Hamilton | Hamilton | Montgomery |
| | Franklin | Cuyahoga | Hamilton | Cuyahoga | Butler | Clark | Summit |
| | Franklin | Clark | Cuyahoga | Cuyahoga | Butler | Butler | Summit |
| | Franklin | Cuyahoga | Mahoning | Cuyahoga | Butler | Butler | Butler |
| Most Popular County (how Popular) (near city) | Franklin County: 7/10 (in Columbus) | Hamilton County: 4/10 (in Cincinnati) | Cuyahoga County: 4/10 (in Cleveland) | Cuyahoga County: 7/10 (in Cleveland) | Butler County: 8/10 (in Cincinnati) | Butler County: 5/10 (in Cincinnati) | Summit County: 5/10 (in Akron) |
| LISA_I (P-value < 0.05) | 22.713 | 10.792 | 15.508 | 15.683 | 33.174 | 26.137 | 19.071 |
| | 7.835 | 6.930 | 15.091 | 13.258 | 28.665 | 17.406 | 18.977 |
| | 5.897 | 6.305 | 15.000 | 12.516 | 13.559 | 17.028 | 14.672 |
| | 5.316 | 5.288 | 13.030 | 11.431 | 11.540 | 16.645 | 13.514 |
| | 5.052 | 5.068 | 11.734 | 10.880 | 11.525 | 13.854 | 12.387 |
| | 4.521 | 4.610 | 11.374 | 10.306 | 11.471 | 13.344 | 11.337 |
| | 4.203 | 4.243 | 10.623 | 7.317 | 11.382 | 11.492 | 11.096 |
| | 3.846 | 3.745 | 9.057 | 7.185 | 11.199 | 9.197 | 10.254 |
| | 3.716 | 3.654 | 8.903 | 7.074 | 6.632 | 8.781 | 9.744 |
| | 3.447 | 3.499 | 8.203 | 6.606 | 6.565 | 8.055 | 9.102 |

**Table S 2. List of top 10 census tracts and their corresponding county by statistics value from 2010 to 2016.** Three large cities: Cleveland, Columbus and Cincinnati, experienced more clustering of opioid poisoning than any other regions.

| Representative terms (from health condition) |
|---|
| Acute Respiratory Failure |
| Alcohol Abuse |
| Anoxic Encephalopathy |
| Anxiety |
| Bipolar Disorder |
| Cardiomegaly |
| Cardiovascular Disease |
| Chronic Back Pain |
| Chronic Obstructive Lung Disease |
| Chronic Obstructive Pulmonary Disease |
| Chronic Urinary Tract Infections, Bi-Polar Disorder, Multiple Abdominal Surgeries, Psoriasis |
| Cirrhosis |
| Cocaine Abuse |
| Coronary Artery Disease |
| Depression |
| Diabetes Mellitus |
| Dilated Cardiomyopathy |
| Drug Abuse |
| Emphysema |
| Exposure To A Cold Environment |
| Extensive Pulmonary Thromboembolism With Polarizable Foreign Material Consistent With Recent And Remote Iv Drug Abuse |
| Fatty Liver |
| Hepatitis C |
| Morbid Obesity |
| Pneumonia |
| Pulmonary Edema |
| Seizure Disorder, Drug And Alcohol Abuse, Remote Traumatic Brain Injury |
| Sleep Apnea |
| Thrombosis Of Right Common Iliac Artery With Attached, Occlusive Thrombus Of Greenfield Type Filter Of Inferior Vena Cava |
| Tobacco Smoke Exposure-Related Lung Disease |

**Table S 3. Results of clustering health conditions. We set the number of clusters to 70 through several empirical experiments**

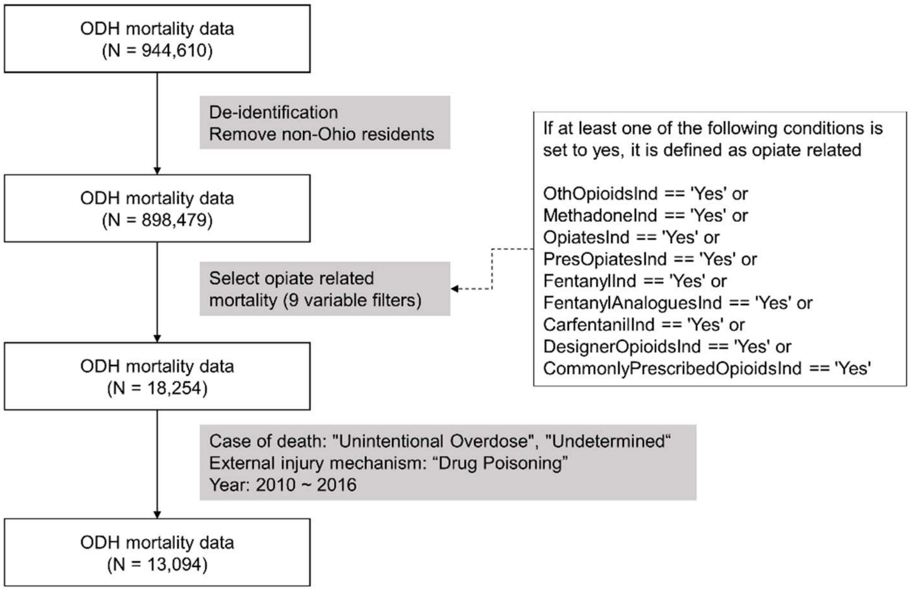


**Figure S1. The process of data refinement.** From the entire ODH mortality data, we finally extracted 13,094 records related with opioid poisoning.

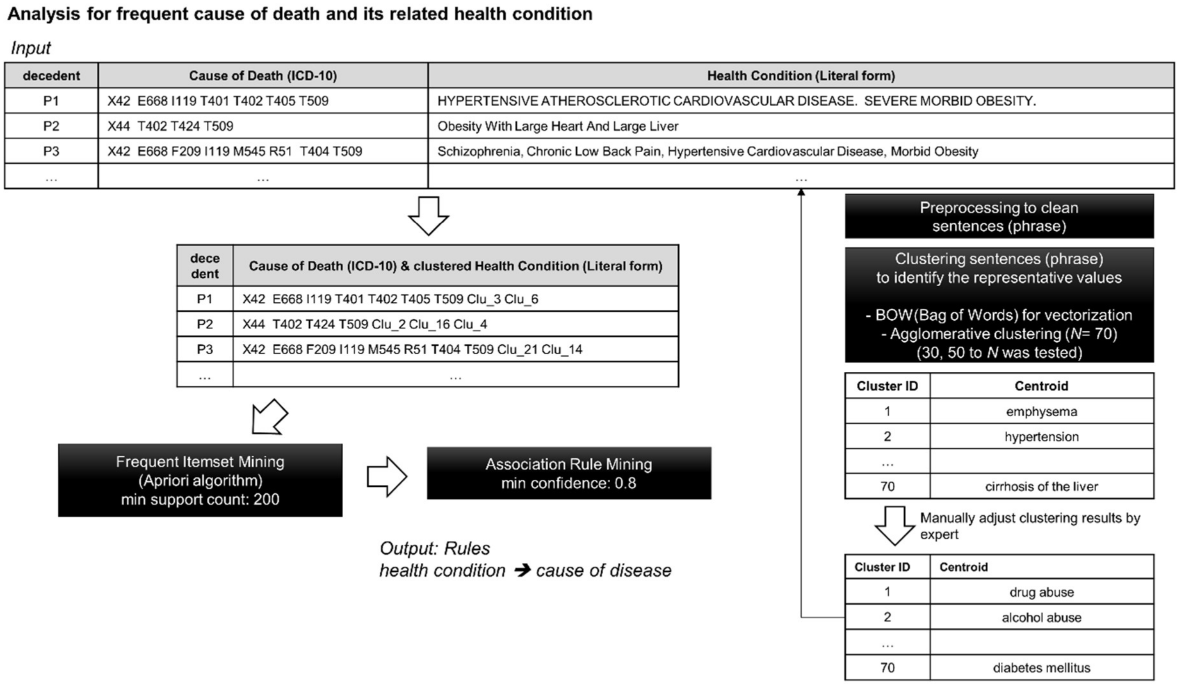


**Figure S2. Entire workflow of co-occurrence analysis with the list of cause of death and corresponding health condition.**
